# Diagnosis of hypertension: Ambulatory pediatric American Heart Association/European Society of Hypertension versus blood pressure load thresholds

**DOI:** 10.1111/jch.14368

**Published:** 2021-10-20

**Authors:** Ajay P. Sharma, Luis Altamirano‐Diaz, Mohamed Mohamed Ali, Katryna Stronks, Amrit Kirpalani, Guido Filler, Kambiz Norozi

**Affiliations:** ^1^ University of Western Ontario London Ontario Canada; ^2^ Division of Nephrology London Health Sciences Centre London Ontario Canada; ^3^ Department of Pediatrics London Health Sciences Centre London Ontario Canada; ^4^ Division of Cardiology London Health Sciences Centre London Ontario Canada; ^5^ Department of Pediatric Cardiology and Intensive care Medical school Hannover Hanover Germany

**Keywords:** ambulatory blood pressure monitoring, hypertension diagnosis, masked hypertension, pediatric blood pressure, pediatric hypertension, white coat hypertension

## Abstract

The agreement between the traditionally‐used ambulatory blood pressure (ABP)‐load thresholds in children and recently‐recommended pediatric American Heart Association (AHA)/European Society of Hypertension (ESH) ABP thresholds for diagnosing ambulatory hypertension (AH), white coat hypertension (WCH), and masked hypertension (MH) has not been evaluated. In this cross‐sectional study on 450 outpatient participants, the authors evaluated the agreement between previously used ABP‐load 25%, 30%, 40%, 50% thresholds and the AHA/ESH thresholds for diagnosing AH, WCH, and MH. The American Academy of Pediatrics thresholds were used to diagnose office hypertension. The AHA threshold diagnosed ambulatory normotension/hypertension closest to ABP load 50% in 88% (95% CI 0.79, 0.96) participants (k 0.67, 95% CI 0.59, 0.75) and the ESH threshold diagnosed ambulatory normotension/hypertension closest to ABP load 40% in 86% (95% CI 0.77, 0.94) participants (k 0.66, 95% CI 0.59, 0.74). In contrast, the AHA/ESH thresholds had a relatively weaker agreement with ABP load 25%/30%. Therefore, the diagnosis of AH was closest between the AHA threshold and ABP load 50% (difference 3%, 95% CI ‐2.6%, 8.6%, *p* = .29) and between the ESH threshold and ABP load 40% (difference 4%, 95% CI ‐2.1%, 10.1%, *p* = .19) than between the AHA/ESH and ABP load 25%/30% thresholds. A similar agreement pattern persisted between the AHA/ESH and various ABP load thresholds for diagnosing WCH and MH. The AHA and ESH thresholds diagnosed AH, WCH, and MH closest to ABP load 40%/50% than ABP load 25%/30%. Future outcome‐based studies are needed to guide the optimal use of these ABP thresholds in clinical practice.

## 1 INTRODUCTION

The pediatric American Heart Association (AHA) and European Society of Hypertension (ESH) guidelines recommend the use of 24‐h ambulatory blood pressure (ABP) monitoring (ABPM) to diagnose ambulatory hypertension (AH).^1^, ^2^, ^3^ Based on office hypertension/normotension as per an office blood pressure (OBP) threshold, AH as per an ABP threshold diagnoses white coat hypertension (WCH) and masked hypertension (MH).^1^, ^2^, ^3^, ^4^, ^5^

In children, ABP load (proportion of ABP readings higher than 95^th^ mean ABP percentile) has been traditionally used to diagnose AH.^6^, ^7^, ^8^, ^9^, ^10^, ^11^ However, the lack of consensus and paucity of outcome‐based studies have led to the use of various ABP load thresholds, ranging from 25%,^8^, ^11^ 30%,^12^ 40%^13^ to 50%.^10^ To establish a uniformity in the use of ABP thresholds and consistency with the use of mean‐ABP thresholds in adults,^14^, ^15^ the pediatric AHA and ESH guidelines have recently recommended the use of 24‐h mean ABP‐based thresholds to diagnose AH (AHA threshold: 24‐h mean systolic/diastolic ABP ≥95^th^ ABP percentiles along with 24‐h ABP systolic/diastolic load ≥25%^1^, ^3^; ESH threshold: 24‐h mean systolic/diastolic 95^th^ ABP percentiles or ≥130/80 mm Hg if 24‐h mean systolic/diastolic 95^th^ ABP percentile exceeds 130/80 mm Hg^2^).

The agreement between various ABP‐load thresholds and the AHA/ESH thresholds is not known, which makes it challenging to interpret the diagnosis of AH across the studies using either AHA/ESH or an ABP‐load threshold. Therefore, we evaluated the agreement between the AHA/ESH thresholds and previously used ABP‐load thresholds (25%, 30%, 40% and 50%) for diagnosing AH, WCH, and MH.

## 2 METHODS

This was a single center, retrospective cross‐sectional study performed after approval by the University of Western Ontario research ethics board. The study involved a retrospective review of existing clinical data and was therefore exempted from the need for an individual informed consent. The records of children who underwent 24‐h ABPM at a tertiary care outpatient hypertension clinic (London, Ontario, Canada) were collected. The participants were referred to our outpatient clinic because of suspected hypertension based on OBP assessments by the primary health care providers. In those with multiple ABPM assessments, their first ABPM recording was included for this analysis. The data was collected between January, 2003 and December, 2008 (*n* = 159) as a part of previous studies^16^, ^17^, ^18^, ^19^ and recently between January, 2018 and September, 2020 (n = 291). During both the study periods, there was a uniformity in the protocol regarding offering ABPM to patients older than 5 years and for evaluating secondary hypertension (Fourth Report guidelines^20^ during the first study period and similar recommendations by the American Academy of Pediatrics‐AAP‐guidelines during the second period^1^). The participants with an inadequate ABPM or missing OBP recordings were excluded. Anthropometric measurements (height, measured by stadiometer; weight, measured using a high‐precision industrial scale) and chronological age (calculated from the difference between the date of the appointment and the date of birth) were obtained as a clinical routine. Body mass index (BMI) percentiles were calculated based on the *Centers for Disease Control and Prevention* reference intervals (overweight: 85th–95th percentiles; obese: > 95th percentile).^21^

### 2.1 Office blood pressure (OBP) measurement

We performed OBP measurements by the methodology recommended by the Fourth report and the AAP guidelines.^1^, ^20^ A trained nurse measured OBP in a quiet room with child seated for 3–5 min, back supported and feet uncrossed on the floor. OBP was measured in the right arm, with the arm at the heart level, using an appropriate‐sized cuff. Cuff size was selected according to child's upper right arm as recommended by the Fourth report and AAP guidelines,^1^, ^20^ making sure that bladder length covers 80%–100% and width 40% of the mid arm circumference. OBP was initially measured by a calibrated oscillometric device (V 100, Dinamap, Tampa, FL, USA).^22^ If oscillometric OBP measurements remain elevated (≥90th percentile), auscultatory OBP measurements were performed using a calibrated aneroid sphygmomanometer, with an appropriate‐sized cuff as described for oscillometric measurements.^1^, ^20^ An average of last two auscultatory OBP measurements was used to diagnose office hypertension.^1^, ^20^

### 2.2 Ambulatory blood pressure measurement (ABPM)

24‐h ABPM was performed with an oscillometric ambulatory BP monitors (model 90207 Space‐labs, Inc, Redmond, WA, USA).^23^, ^24^ A trained nurse chose an appropriate‐sized cuff and conducted ABPM as per the recommendations by the Fourth report and AAP guidlines.^1^, ^20^ The cuff was placed on the nondominant arm, with ABP recordings planned for every 20 min during day and 30 min during night.^1^, ^3^ The participants were instructed to continue with their regular daily activity, to avoid strenuous exercise, to keep arm still at the time of ABP recording by the equipment and to maintain a wake‐sleep log for defining day and night ABP. The data was inspected to edit outliers. The adequacy of ABP recordings was established based on minimum one reading per hour during day and nighttime, and more than 40 readings in 24‐h.^1^ 24‐h, day and night systolic and diastolic ABP were analyzed by the 24‐h, day and night mean 95^th^ systolic and diastolic ABP percentiles as per the normative data by Wuhl and coworkers^24^ Systolic and diastolic ABP load over 24‐h, day and night were calculated as the percentage of ABP measurements higher than respective mean 95^th^ systolic and diastolic ABP percentiles.^24^

### 2.3 Outcomes

Our primary outcome was to evaluate the agreement between the AHA/ESH thresholds and various ABP‐load thresholds in earlier studies for diagnosing AH. Our secondary outcome was to assess the agreement between the AHA/ESH thresholds and the ABP‐load thresholds for diagnosing WCH/MH, with office normotension/hypertension diagnosed by the AAP threshold.^1^

### 2.4 Definitions

OBP was diagnosed as per the OBP threshold recommended by the AAP guidelines.^1^ AH was diagnosed individually based on the AHA threshold, ESH threshold and ABP loads 25%, 30%, 40%, 50% thresholds.^8^, ^10^, ^11^, ^12^ Office normotension/hypertension and ambulatory normotension/hypertension status were used to diagnose normotension, WCH, MH and hypertension (thresholds and definitions summarized in Table 1).

**TABLE 1:** Office blood pressure threshold, ambulatory blood pressure thresholds and definitions for diagnosing hypertension

Office blood pressure (OBP) threshold
AAP threshold	For < 13 years: age‐, sex‐, and height‐specific systolic or diastolic ≥95^th^ OBP percentiles; for ≥13 year ≥130/80 mm Hg.^1^

Ambulatory blood pressure (ABP) thresholds
24‐h mean 95^th^ ABP percentile	Age‐, sex‐, height‐ specific mean 24‐h systolic or diastolic ≥95^th^ ABP percentiles as per normative ABP thresholds recommended by *Wuhl* and coworkers.^5^
AHA threshold	For < 16 years‐24‐h mean systolic or diastolic ABP ≥ 95^th^ ABP percentiles along with systolic or diastolic ABP load ≥25% based on 24‐h mean 95^th^ ABP percentile.^1^ For ≥ 16 years: ABP threshold 130/80 mm Hg and ABP load ≥25%.
ESH threshold	24‐h mean systolic or diastolic ABP ≥ 95^th^ ABP percentiles or 24‐h systolic or diastolic ABP ≥130/80 mm Hg if 24‐h mean 95^th^ ABP percentiles exceed130/80 mm Hg^2^
ABP load 25%	24‐h mean systolic or diastolic ABP > 25% readings above 24‐h mean systolic or diastolic 95^th^ ABP percentile
ABP load 30%	24‐h mean systolic or diastolic ABP > 30% readings above 24‐h mean systolic or diastolic 95^th^ ABP percentile
ABP load 40%	24‐h mean systolic or diastolic ABP > 40% readings above 24‐h mean 95^th^ systolic or diastolic ABP percentile
ABP load 50%	24‐h mean systolic or diastolic ABP > 50% readings above 24‐h mean 95^th^ systolic or diastolic ABP percentile

Definitions for diagnosing hypertension		
Office hypertension	Age < 13 year – Patient's systolic or diastolic OBP ≥ 95^th^ OBP threshold^1^	Age ≥13 year – Patient's systolic OBP ≥130 mm Hg or diastolic OBP ≥80 mm Hg^1^
Ambulatory hypertension	Patient's 24‐h systolic or diastolic ABP diagnosed as ambulatory hypertension, as per the listed ABP thresholds	
Normotension	No office hypertension	No ambulatory hypertension
White coat hypertension	Office hypertension	Ambulatory normotension
Masked hypertension	Office normotension	Ambulatory hypertension
Hypertension	Office hypertension	Ambulatory normotension

*Abbreviations*: AAP, American Academy of Pediatrics; OBP, Office blood pressure; ABP, Ambulatory blood pressure; AHA, American Heart Association; ESH, European Society of Hypertension.

### 2.5 Statistical methods

Normally distributed continuous variables were reported as mean (standard deviation), otherwise as median (interquartile range). Categorical variables were reported as frequency and percentage. Continuous variables were compared with the parametric unpaired *t* test or the non‐parametric Mann‐Whitney U test, as appropriate. Categorical variables were compared with chi‐square test. Systolic and diastolic OBP z‐scores and 95^th^ OBP percentiles were calculated based on the OBP references using the computation methodology recommended by the AAP guidelines.^1^, ^25^ 24‐h systolic and diastolic ABP z‐score and 95^th^ ABP percentiles were calculated based on the ABP references by Wuhl and coworkers using Box‐Cox transformations with age‐sex‐specific estimates of the distribution median, coefficient of variation, and degree of skewness.^24^ The agreement between the AHA/ESH thresholds and ABP‐load thresholds was calculated by the accuracy (the proportion of ABP classified similarly by the two ABP thresholds) and kappa statistics.^26^ Given the fact that AHA threshold is based on 24‐h mean ABP 95^th^ percentile and ABP load ≥ 25% estimated by 24‐h mean ABP 95^th^ percentile, we limited our analysis on the agreement between the AHA/ESH thresholds and ABP load thresholds based on 24‐h mean ABP 95^th^ percentile. For an age‐based analysis, adolescents were defined as those with age ≥ 13 years, as recommended by the AAP guidelines.^1^ Accuracy and Kappa statistics were calculated on Medcalc version 18.11. (MedCalc Software bvba, Mariakerke, Belgium). All other statistical analysis was performed IBM SPSS Statistics for Windows, version 25.0. (IBM Corp., Armonk, NY, USA).

## 3 RESULTS

### 3.1 Patient characteristics

In the initial screening, 544 participants who had ABPM studies during the recruitment period met the inclusion criteria. Ninety‐four participants were excluded for the following reasons: 53 had less than two OBP recordings and 41 had an inadequate ABPM. Four hundred and fifty eligible participants aged 5–18 years with complete ABPM and OBP recordings were included in this analysis. The study sample included with 59% adolescents ≥ 13 years, 41% females and 55% overweight/obese participants. Each participant was included with a single ABPM recording in the analysis. AHA threshold diagnosed AH in 26% (95% CI 0.21, 0.31), ESH threshold in 30% (95% CI 0.25, 0.35), ABP loads 25% in 53% (95% CI 0.46, 0.60), 30% in 46% (95% CI 0.39, 0.52), 40% in 34%, (95% CI 0.28, 0.39) and 50% in 23% (95% CI 0.18, 0.28) participants. AH by the ABP‐load thresholds did not significantly differ in age, sex, BMI z‐score, the proportion of adolescents, overweight/obese participants, and those with office hypertension, primary hypertension and not taking a blood pressure medication (Table 2).

**TABLE 2:** Patient characteristics

	Entire group (*n* = 450)	Ambulatory hypertension by ABP load 25% (*n* = 239)	Ambulatory hypertension by ABP load 30% (*n* = 205)	Ambulatory hypertension by ABP load 40% (*n* = 153)	Ambulatory hypertension by ABP load 50% (*n* = 104)
Mean age years (SD)	13.03(3.58)	13.33 (3.51)	13.46(3.53)	13.36(3.63)	13.46 (3.70)
Age ≥13 years (%)	267 (59%)	145 (60%)	126 (61%)	92 (60%)	61 (59%)
Females (%)	183 (41%)	103 (43%)	88 (43%)	67 (44%)	41 (39%)
Overweight/obese (%)	248 (55%)	138 (58%)	117 (57%)	84 (55%)	56 (54%)
BMI z‐score (IQR)	1.21 (0.16,2.00)	1.41 (0.33,2.13)	1.42 (0.32, 2.19)	1.43 (0.33, 2.21)	1.42 (0.36,2.19)
Office hypertension (%)	57 (36%)	156 (65%)	136 (66%)	108 (71%)	76 (73%)
OBP systolic z score (IQR)	1.68 (0.33,2.30)	2.06 (1.29,2.78)	2.12 (1.33, 2.89)	2.18 (1.46, 2.87)	2.20 (1.60,2.97)
OBP diastolic z‐score (IQR)	0.60 (‐0.05,1.41)	0.85 (0.27,1.51)	0.84 (0.27,1.53)	1.02 (0.32,1.57)	1.19 (0.34,1.74)
Primary hypertension	352 (78%)	201 (84%)	172 (84%)	132 (86%)	90 (87%)
Secondary hypertension	98 (22%)	38 16%)	33 (16%)	21 (14%)	14 (13%)
No BP medication	322 (72%)	186 (78%)	161 (79%)	122 (80%)	88 (85%)
BP Medication	128 (28%)	53 (22%)	44 (21%)	31 (20%)	16 (15%)
ABP systolic z‐score (IQR)	0.32 (‐0.60,1.18)	1.07 (0.41,1.90)	1.18 (0.63,2.06)	1.52 (0.86, 2.34)	1.94 (1.13,2.77)
ABP systolic load % (IQR)	21.42 (7.14, 44.44)	42.20 (28.24,62.75)	46.15 (32.46,66.67)	55.50 (44.10,74.54)	66.53 (54.78, 85.29)
ABP diastolic z‐score (IQR)	0.13 (‐0.69,1.18)	1.04 (0.12,1.88)	1.18 (0.24,1.98)	1.40 (0.40,2.34)	1.88 (0.81,2.79)
ABP diastolic load % (IQR)	15.38 (6.67, 34.20)	32.20 (17.65,48.36)	35.48 (18.11,51.44)	41.60 (22.10,56.60)	51.18 (31.66,71.70)

*Abbreviations*: SD, Standard deviation; IQR, Interquartile range; BMI, Body mass index; BP, Blood pressure; OBP, Office blood pressure; ABP, Ambulatory blood pressure.

*Definitions*: Office hypertension was diagnosed by the American Academy of Pediatrics threshold^1^. ABP load‐ the percentage of ABP measurements that exceeded mean 24‐h 95^th^ ABP percentile according to the ABPM references.^5^

### 3.2 Agreement between the AHA/ESH and ABP‐load thresholds for diagnosing ambulatory normotension and hypertension

#### 3.2.1 Agreement between the AHA and ABP‐load thresholds

**FIGURE 1:**
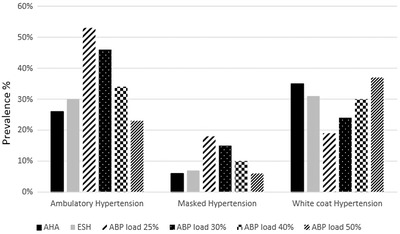
Ambulatory hypertension, masked hypertension and white coat hypertension diagnosed by the American Heart Association (AHA), European Society of Hypertension (ESH) and 24‐h ambulatory blood pressure (ABP) load 25%, 30%, 40%, and 50% thresholds; office hypertension diagnosed by the American Academy of Pediatrics threshold

Among all ABP‐load thresholds, the AHA threshold diagnosed ambulatory normotension/hypertension closest to ABP load 50% in 88% (95% CI 0.79, 0.96) participants (k 0.67, 95% CI 0.59, 0.75) and ABP load 40% in 86% (95% CI 0.77, 0.95) participants (k 0.67, 95% CI 0.60, 0.74). In contrast, the AHA threshold had a lower agreement to diagnose ambulatory normotension/hypertension with ABP load 30% in 80% (95% CI 0.71, 0.88) participants (k 0.57, 95% CI 0.50, 0.64) and ABP load 25% in 73% (95% CI 0.65, 0.81) participants (k 0.47, 95% CI 0.40, 0.54) (Table 3). Therefore, AH by the AHA threshold was closest to that by the ABP load 50% (difference 3%, 95% CI ‐2.6%, 8.6%, *p* = .29) and ABP load 40% (difference 8%, 95% CI 2%, 13.9%, *p* = .00) than that with ABP load 30% (difference 20%, 95% CI 13.8%, 26%, *p* < .001) and ABP load 25% (difference 27%, 95% CI 20.7%, 30%, *p* < .001) (Table 4) (Figure 1). The agreement between the AHA threshold and different ABP load thresholds remained consistent in sub‐groups based on age, sex, primary hypertension, and those not taking blood pressure medications (Table 4).

**TABLE 3:** Agreement between the AHA/ESH thresholds and ABP load 25%, 30%, 40% and 50% thresholds, with office normotension/hypertension diagnosed by the AAP threshold

	AHA threshold		ESH threshold	
	Accuracy^a^% (95^th^ CI)	Kappa (95% CI)	Accuracy% (95^th^ CI)	Kappa (95% CI)
	**ABP load 25%**			
Entire group (*n* = 450)	73% (0.65, 0.81)	0.47 (0.40, 0.54)	74% (0.66, 0.82)	0.49 (0.42, 0.56)
Age ≥13 years (%) (*n* = 267)	67% (0.57, 0.78)	0.37(0.29, 0.46)	70% (0.61,0.81)	0.43(0.34, 0.52)
Females (%) (*n* = 183)	68% (0.56,0.81)	0.40(0.30, 0.50)	72% (0.60, 0.85)	0.46(0.36,0.57)
Primary hypertension (*n* = 352)	72% (0.63, 0.81)	0.48 (0.40, 0.56)	73% (0.64, 0.82)	0.48 (0.40, 0.56)
No BP medication (*n* = 322)	72% (0.63, 0.82)	0.48 (0.40, 0.56)	73% (0.63, 0.82)	0.48 (0.39, 0.56)
Primary hypertension and No BP medication (*n* = 295)	73% (0.63, 0.82)	0.48 (0.40, 0.56)	73% (0.63, 0.83)	0.48 (0.39, 0.56)
	**ABP load 30%**			
Entire group (*n* = 450)	80% (0.71,0.88)	0.57 (0.50, 0.64)	81% (0.72,0.89)	0.59 (0.52, 0.67)
Age ≥13 years (%) (*n* = 267)	74% (0.64, 0.85)	0.47(0.38, 0.56)	77% (0.67, 0.89)	0.54(0.45,0.63)
Females (%) (*n* = 183)	75% (0.63, 0.89)	0.49(0.38, 0.61)	79% (0.66, 0.93)	0.57(0.46, 0.68)
Primary hypertension (*n* = 352)	80% (0.70,0.89)	0.58 (0.50, 0.67)	80% (0.71,0.90)	0.59 (0.50, 0.68)
No BP medication (*n* = 322)	79% (0.69, 0.89)	0.58 (0.50, 0.66)	79% (0.70,0.89)	0.59 (0.50, 0.67)
Primary hypertension and no BP medication (*n* = 295)	79% (0.69, 0.90)	0.58 (0.50, 0.67)	79% (0.69, 0.90)	0.59 (0.50, 0.68)
	**ABP load 40%**			
Entire group (*n* = 450)	86% (0.77, 0.95)	0.67 (0.60, 0.74)	86% (0.77, 0.94)	0.66 (0.59, 0.74)
Age ≥13 years (%) (*n* = 267)	83% (0.72, 0.95)	0.60(0.49, 0.70)	83%(0.73, 0.95)	0.62(0.52, 0.72)
Females (%) (*n* = 183)	83% (0.70, 0.97)	0.62(0.50, 0.73)	85% (0.72, 0.99)	0.66 (0.55, 0.78)
Primary hypertension (*n* = 352)	85% (0.75, 0.95)	0.67 (0.59, 0.75)	84% (0.74, 0.92)	0.63 (0.54, 0.72)
No BP medication (*n* = 322)	85% (0.75, 0.95)	0.66 (0.58, 0.75)	82% (0.73, 0.93)	0.63 (0.54, 0.71)
Primary hypertension and no BP medication (*n* = 295)	84% (0.74, 0.95)	0.66 (0.57, 0.75)	83% (0.72, 0.94)	0.63 (0.54, 0.72)
	**ABP load 50%**			
Entire group (*n* = 450)	88% (0.79, 0.96)	0.67 (0.59, 0.75)	86% (0.77, 0.95)	0.64 (0.57, 0.72)
Age ≥13 years (%) (*n* = 267)	86% (0.76 0.98)	0.621(0.50, 0.73)	85% (0.75 0.97)	0.62(0.51, 0.73)
Females (%) (*n* = 183)	87% (0.74,1.02)	0.66(0.53, 0.79)	88% (0.75, 1.03)	0.70(0.58,0.81)
Primary hypertension (*n* = 352)	86% (0.76, 0.96)	0.66 (0.57, 0.74)	84% (0.74, 0.92)	0.61 (0.51, 0.70)
No BP medication (*n* = 322)	86% (0.76, 0.96)	0.65 (0.56, 0.74)	83% (0.73, 0.94)	0.61 (0.52, 0.71)
Primary hypertension and no BP medication (*n* = 295)	85% (0.75, 0.96)	0.64 (0.55, 0.74)	83% (0.72, 0.94)	0.61 (0.51, 0.70)

*Definitions*: AHA threshold: 24‐h mean systolic or diastolic ABP ≥95^th^ ABP percentile and 24‐h systolic or diastolic ABP load ≥25% ^1^. ESH threshold: 24‐h systolic or diastolic ABP ≥95^th^ ABP percentile or ABP≥130/80 mm Hg (if 24‐h mean systolic or diastolic ABP 95^th^ percentile ≥130/80 mm Hg)^2^; ABP load 25%, 30%, 40% and 50% thresholds: 24‐h systolic or diastolic ABP load > than these systolic or diastolic ABP loads; ABP load: proportion of systolic and diastolic ABP readings higher than 24‐h mean 95^th^ ABP systolic and diastolic percentiles. 24‐h mean systolic and diastolic ABP 95^th^ percentiles were calculated based on the ABP references by Wuhl and coworkers^5^.

*Abbreviations*: ABP, Ambulatory blood pressure; CI, Confidence interval; AHA, American Heart Association; ESH, European Society of Hypertension; BP, Blood pressure; AAP, American Academy of Pediatrics.

^a^ Accuracy‐the proportion of ABP classified similarly by both the ABP thresholds into ambulatory normotension/hypertension.

**TABLE 4:** Diagnosis of ambulatory hypertension, white coat hypertension and masked hypertension based on the ambulatory blood pressure (ABP) thresholds and office normotension/hypertension diagnosed by the American Academy of Pediatrics (AAP) threshold

	AHA threshold (95% CI)	ESH threshold (95% CI)	ABP load 25% (95% CI)	ABP load 30% (95% CI)	ABP load 40% (95% CI)	ABP load 50% (95% CI)
**Ambulatory hypertension (95% CI)**						
Entire group (*n* = 450)	26% (0.21, 0.31)	30% (0.25, 0.35)	53% (0.46, 0.60)	46% (0.39, 0.52)	34% (0.28, 0.39)	23% (0.18, 0.28)
Age ≥13 years (*n* = 267)	21% (0.16,0.28)	27% (0.21, 0.34)	54% (0.45, 0.63)	47% (0.39, 0.56)	34% (0.27, 0.42)	23% (0.17, 0.29)
Females (*n* = 183)	24% (0.17, 0.32)	30% (0.22, 0.38)	56% (0.45, 0.68)	48% (0.38,0.59)	36% (0.28, 0.46)	22% (0.16, 0.30)
Primary hypertension (*n* = 352)	29% (0.24, 0.35)	34% (0.28, 0.40)	57% (0.49 0.65)	49% (0.41, 0.56)	37% (0.31, 0.44)	26% (0.20, 0.31)
No BP medication (n = 322)	30% (0.24, 0.36)	35% (0.28, 0.42)	58% (0.49, 0.66)	50% (0.42 0.58)	38% (0.31, 0.45)	27% (0.21, 0.33)
Primary hypertension and no BP medication (*n* = 295)	30% (0.24, 0.37)	35% (0.29, 0.43)	58% (0.49,0.67)	50% (0.42, 0.58)	38% (0.31, 0.46)	27% (0.21, 0.34)
**White coat hypertension (95% CI)**						
Entire group (*n* = 450)	35% (0.29, 0.40)	31% (0.25, 0.36)	19% (0.15, 0.24)	24% (0.19, 0.28)	30% (0.25, 0.35)	37% (0.31, 0.43)
Age ≥13 years (*n* = 267)	36% (0.29, 0.43)	31% (0.24,0.38)	17% (0.12, 0.23)	22% (0.16, 0.28)	29% (0.23, 0.36)	37% (0.29, 0.44)
Females (*n* = 183)	34% (0.26,0.44)	30% (0.22, 0.39)	14% (0.09, 0.20)	20% (0.14, 0.27)	28% (0.20, 0.36)	36% (0.27, 0.45)
Primary hypertension (*n* = 352)	36% (0.30, 0.43)	32% (0.26,0.38)	21% (0.16, 0.26)	26% (0.20, 0.31)	32% (0.26, 0.38)	40% (0.33, 0.47)
No BP medication (n = 322)	37% (0.31, 0.44)	33% (0.27, 0.40)	21% (0.16, 0.27)	26% (0.21, 0.32)	33% (0.26, 0.39)	40% (0.33 0.47)
Primary hypertension and no BP medication (*n* = 295)	37% (0.30, 0.45)	33% (0.26, 0.40)	22% (0.16, 0.27)	27% (0.21, 0.33)	33% (0.26, 0.40)	41% (0.33, 0.48)
**Masked hypertension (95% CI)**						
Entire group (*n* = 450)	6% (0.04, 0.09)	7% (0.04, 0.09)	18% (0.14, 0.22)	15% (0.11, 0.19)	10% (0.07, 0.13)	6% (0.04, 0.08)
Age ≥13 years (*n* = 267)	6% (0.03, 0.10)	7% (0.04, 0.11)	20% (0.15, 0.26)	17% (0.12, 0.23)	12% (0.08, 0.17)	8% (0.05, 0.12)
Females (*n* = 183)	6% (0.03, 0.10)	6% (0.03, 0.11)	17% (0.11,0.24)	15% (0.10, 0.22)	11% (0.07, 0.17)	5% (0.02, 0.10)
Primary hypertension (*n* = 352)	6% (0.04, 0.09)	7% (0.04, 0.10)	19% (0.14, 0.23)	15% (0.11, 0.20)	10% (0.07, 0.14)	6% (0.04, 0.09)
No BP medication (*n* = 322)	8% (0.05, 0.12)	9% (0.06, 0.12)	20% (0.15, 0.25)	17% (0.12, 0.22)	11% (0.08, 0.15)	8% (0.05, 0.11)
Primary hypertension and no BP medication (*n* = 295)	7% (0.04, 0.11)	8% (0.05, 0.12)	19% (0.14, 0.25)	16% (0.12, 0.21)	11% (0.07, 0.15)	7% (0.04, 0.11)

*Abbreviations*: AAP, American Academy of Pediatrics; ABP, Ambulatory blood pressure; CI, Confidence interval; BP, Blood pressure; AHA, American Heart Association; ESH, European Society of Hypertension.

*Definitions*: AHA threshold‐ 24‐h systolic or diastolic ABP ≥95^th^ ABP percentile and 24‐h systolic or diastolic ABP load ≥25% ^1^; ESH threshold‐ 24‐h systolic or diastolic mean ABP ≥95^th^ ABP percentile or ABP≥130/80 mm Hg (if 24‐h mean systolic or diastolic ABP 95^th^ percentile ≥130/80 mm Hg)^2^; ABP load 25%, 30%, 40% and 50% thresholds‐ Systolic or diastolic ABP load higher than these systolic or diastolic ABP loads; ABP load: proportion of systolic or diastolic ABP readings higher than 24‐h mean systolic or diastolic 95^th^ ABP percentiles. 24‐h mean systolic and diastolic ABP 95^th^ percentile was calculated based on the ABP references by Wuhl and coworkers^5^; AAP threshold‐ Age‐sex‐height specific systolic/diastolic office blood pressure ≥95^th^ percentile as per the AAP guidelines^1^ ^.^

#### 3.2.2 Agreement between the ESH and ABP‐load thresholds

Similar to the AHA threshold, the ESH threshold maintained a stronger agreement with ABP load 40% in 86% (95% CI 0.77, 0.94) participants (k 0.66, 95% CI 0.59, 0.74) and ABP load 50% in 86% (95% CI 0.77, 0.95) participants (k 0.64, 95% CI 0.57, 0.72) than with ABP load 30% in 81% (95% CI 0.72, 0.89) participants (k 0.59, 95% CI 0.52, 0.67) and ABP load 25% in 74% (95% CI 0.66, 0.82) participants (k 0.49, 95% CI 0.42, 0.56) (Table 3). In contrast to the AHA threshold, the AH by the ESH threshold was relatively closer with ABP load 40% (difference 4%, 95% CI ‐2.1%, 10.1%, *p* = .19) than with ABP load 50% (difference 7%, 95% CI 1.2%, 12.7%, *p* = .01) (Table 4), (Figure 1). The difference in the diagnosis of AH between the ESH and ABP load 30% (difference 16%, 95% CI 4.7%, 22.1%, *p* < .001) and ABP load 25% (difference 23%, 95% CI 16.6%, 29%, *p* < .001) continued to be larger than that with the ABP load 40%/50%. The agreement between the ESH threshold and different ABP load thresholds remained consistent in sub‐group analysis based on age, sex, primary hypertension and those not on blood pressure medications (Table 3).

### 3.3 Diagnosis of WCH/MH by the AHA/ESH and ABP‐load thresholds

The AHA threshold diagnosed WCH in 35% participants, which was closely associated with that by the ESH threshold in 31% participants (difference 4%, 95% CI ‐2.1%, 10.1%, *p* = .20) (Table 4), (Figure 1). The AHA threshold diagnosed WCH closest to the ABP load 50% (*n* = 37%, difference 2%, 95% CI ‐4.3%, 8.2%, *p* = .53) and ABP load 40% (*n* = 30%, difference 5%, 95% CI ‐1.1%, 11.1%, *p* = .10); however, the difference widened between the AHA threshold and ABP load 30% (*n* = 24%, difference 11%, 95% CI 5%, 16.8%, *p* = .00) and ABP load 25% (*n* = 19%, difference 16%, 95% CI 10.2%, 21.6%, *p* = .00). In contrast to the AHA threshold, the diagnosis of WCH by the ESH threshold was relatively closer with ABP load 40% (difference 1%, 95% CI ‐5%, 7%, *p* = .74) than with ABP load 50% (difference 6%, 95% CI ‐0.2%, 12.1%, *p* = .05). Similar to the AHA threshold, the ESH threshold had a larger difference with ABP load 30% (difference 7%, 95% CI 1.2%, 12.8%, *p* = .01) and ABP load 25% (difference 12%, 95% CI 6.4%, 17.5%, *p* < .001) (Table 4). The agreement pattern in the diagnosis of WCH between the AHA/ESH thresholds and the ABP load thresholds did not significantly change when analyzed in the sub‐groups based on age, sex, primary hypertension and those not on blood pressure medications (Table 4).

For the diagnosis of MH, both AHA (*n* = 6%) and ESH (*n* = 7%) thresholds showed a closer association with ABP load 50% (*n* = 6%; AHA: difference 0%, 95% CI ‐3.2%, 3.2%, *p* = 1.00; ESH: difference of 1%, 95% CI ‐2.3%, 4.3%, *p* = .54) (Table 4), (Figure 1). The difference in diagnosis of MH widened between the AHA/ESH thresholds and ABP load 40% (AHA: difference 4%, 95% CI 0.4%, 7.6%, *p* = .02; ESH: difference 3%, 95% CI ‐0.7%, 6.7%, *p* = .10), ABP load 30% (AHA: difference 9%, 95% CI 5%, 13%, *p* < .001; ESH: difference 8%, 95% CI 3.9%, 12.1%, *p* = .00) and ABP load 25% (AHA: difference 12%, 95% CI 7.8%, 16.2%, *p* < .001; ESH: difference 11%, 95% CI 6.7%, 15.3%, *p* < .001) (Table 4). The agreement pattern in the diagnosis of MH by the AHA/ESH thresholds and the ABP load thresholds did not significantly change when analyzed in the subgroups based on age, sex, primary hypertension, and those not on blood pressure medications (Table 4).

## 4 DISCUSSION

In the absence of pediatric literature on the agreement between various ABP‐load thresholds (ABP load 25%, 30%, 40%, and 50%) used in previous studies^6^, ^7^, ^8^, ^9^, ^10^, ^11^ and the recently recommended AHA/ESH thresholds,^1^, ^2^ our findings provide information on the agreement between the AHA/ESH thresholds and ABP load thresholds for diagnosing AH and WCH/MH. We found that the diagnosis of AH, WCH and MH was closer between the AHA/ESH and ABP load 40%/50% than with ABP load 25%/30% thresholds. In relative terms, the diagnosis of AH and WCH was closest between the AHA threshold and ABP load 50%, and the ESH threshold and ABP load 40%. The diagnosis of MH by the AHA and ESH thresholds was closest to ABP load 50%.

The findings from our studies are important in the context of the recent guidelines endorsing a shift from the use of ABP load thresholds in previous pediatric studies^6^, ^7^, ^8^, ^9^, ^10^, ^11^ to mean ABP‐based AHA^1^, ^3^ and ESH^2^ thresholds. The recommendation from the AHA/ESH guidelines is a step towards establishing a consistency with adult hypertension guidelines recommending the use of mean ABP thresholds.^14^, ^15^ Moreover, despite a strong collinearity between the mean ABP and ABP load for predicting target organ damage,^8^, ^27^, ^28^, ^29^, ^30^ mean ABP conceptually represents the extent of ABP elevation whereas ABP load denotes the frequency of ABP elevation. Therefore, in patients with sustained high mean ABP, ABP load close to 100% cease to offer additional information,^31^ whereas in patients with normal mean ABP, ABP load might quantify the degree of ABP fluctuations above normal limits.^6^, ^7^ Consequently, recent studies reevaluated the role of ABP load as an additive covariate to mean ABP, which showed no added increase in the prediction of target organ damage by mean ABP after accounting for ABP load.^8^, ^27^, ^28^ In absence of an additive benefit of ABP load, the recognition of AH in clinical practice requires either a mean ABP or an ABP‐load threshold. In this scenario, our findings provide a connecting link for a consistent interpretation of AH based on either AHA/ESH threshold or one of the ABP‐load thresholds.

The stronger agreement between the AHA/ESH thresholds and ABP load 40%/50% instead of with ABP load 25%/30% can be explained by the fact that the proportion of AH diagnosed by the AHA/ESH thresholds fall between that by the ABP load 40% and 50%. AH by the AHA threshold (*n* = 26%) and ESH threshold (*n* = 30%) was higher than AH by ABP load 50% (n = 23%) and lower than that by ABP load 40% (*n* = 34%). In contrast, ABP load 25% and 30% diagnosed significantly higher AH than the AHA/ESH thresholds in 53% and 46% participants, respectively. Notably, the agreement pattern between the AHA/ESH thresholds and ABP‐load thresholds for diagnosing AH maintained a similar pattern when analyzed separately in adolescents, participants with primary hypertension and those on no antihypertensive medication. Lower threshold level of the ESH threshold than the AHA threshold can explain a relative proximity of the ESH threshold with ABP load 40% and the AHA threshold with the ABP load 50% for diagnosing AH and WCH.

In a previous pediatric study by Koshy and coworkers, mean day and night ABP thresholds showed a stronger agreement with corresponding day and night ABP load 50%, followed by that with ABP load 40% and 30%^32^ for diagnosing AH. Though this study did not evaluate the AHA/ESH thresholds or the diagnosis of WCH/MH, the findings from this study support our observations on a stronger agreement between the mean ABP based AHA/ESH thresholds and ABP load 40%/50% than with ABP load 25%/30% thresholds.

Strengths of our study included the use of a standardized methodology for the OBP/ABP measurements and OBP/ABP interpretation as recommended by the Fourth Report and AAP guidelines.^1^, ^20^ Our study limitations include retrospective study design and unavailability of hypertension‐induced target‐organ damage assessment. Though outcome based studies are lacking at this point with the AHA/ESH threshold, a stronger association between ABP load 50% and hypertension‐induced target‐organ damage in pediatric population supports the possibility of a strong association between the AHA/ESH thresholds and target‐organ damage.^10^ Similarly, in adult population the 10‐year risk of a composite cardiovascular end point associated with the 24‐h mean ABP of 130 mm Hg systolic or 80 mm Hg diastolic was exceeded only by ABP load ≥40.0% systolic or 42.3% diastolic.^27^ Though the Fourth Report OBP thresholds diagnose fewer office hypertension than the AAP thresholds,^33^, ^34^, ^35^, ^36^, ^37^, ^38^ the use of either AAP or Fourth Report OBP threshold has not been found to significantly alter the diagnosis of WCH/MH by the AHA and ESH ABP thresholds.^16^ Though the use of OBP measurements from a single visit in our analysis should not affect our main finding on the association between the AHA/ESH and ABP load thresholds for diagnosing AH, it may potentially influence the diagnosis of office hypertension, therefore the estimation of WCH and MH.^1^, ^20^ However, prior OBP assessments with primary health care providers before the referral to our outpatient clinic may have led to some OBP attenuation because of accommodation effect and regression to mean phenomenon, which may have possibly decreased the confounding effect of single visit OBP measurements in our analysis.^20^ Despite the fact that we included participants from two different time periods to enhance the statistical power of our analysis, a consistent practice for evaluating secondary etiologies of hypertension over the two periods^1^, ^20^ should minimize a potential misclassification into primary/secondary hypertension across the periods. Given the fact that the AHA threshold is based on 24‐h mean ABP 95^th^ percentile, our analysis focused on ABP load thresholds estimated by 24‐h mean ABP 95^th^ percentile cannot comment on day/night ABP load thresholds estimated by day/night mean 95^th^ ABP percentiles. However, considering the assumption that 24‐h mean ABP represents day and night ABP, it is possible that day/night mean ABP load thresholds may demonstrate a similar agreement with the AHA/ESH thresholds as observed with 24‐h ABP load thresholds in our analysis. It should be noted that oscillometric OBP/ABP measurements by the commonly used Dinamap and Spacelab equipments have been found to be more accurate for systolic than for diastolic measurements.^22^, ^23^ Moreover, the commonly used ABP references recommended by Wuhl and coworkers, derived from oscillometric ABP measurements, showed minimal age‐related increase in diastolic values.^24^ Therefore, these limitations should be kept in consideration while interpreting diastolic OBP/ABP measurements in clinical practice and in relation to our findings. Predominant Caucasian ethnicity limits the generalizability of our observations to other ethnicities. In view of the tertiary care setting of our study, our results should be applied to a primary care population with caution.

We conclude that the diagnosis of AH by the AHA threshold remains closest to the ABP load 50% and that of ESH threshold closest to the ABP load 40%. With office hypertension diagnosed by the AAP threshold, the AHA/ESH thresholds diagnose WCH/MH closer to ABP load 40%/50% than with the ABP load 25/30%. Further outcome‐based studies focused the AHA/ESH and ABP load thresholds can further refine the use of these ABP thresholds in clinical practice.

## CONFLICT OF INTEREST

None declared

## AUTHOR CONTRIBUTIONS

It is to state that the work described has not been published before, and it is not under consideration for publication anywhere else. For the manuscript, APS conceived the idea, conducted the statistical analysis, drafted the manuscript and supervised overall development of the manuscript. The authors MA and KS helped in complying the data, assisting in statistical analysis and in drafting the manuscript. The authors LAD, AK, GF and KN revised the manuscript and played an important role in interpreting the results. The publication has been approved by all co‐authors. The corresponding author has had full access to the data in the study and final responsibility for the decision to submit for publication.
